# Risk of all-cause mortality in patients with knee osteoarthritis: A systematic review and meta-analysis of cohort studies

**DOI:** 10.1016/j.ocarto.2024.100541

**Published:** 2024-11-08

**Authors:** Pei-En Kao, Amy Ker

**Affiliations:** a Department of Medical Education, Kaohsiung Chang Gung Memorial Hospital, Kaohsiung 833401, Taiwan; b School of Medicine, Chung Shan Medical University, Taichung, Taiwan

**Keywords:** All-cause mortality, Mortality, Knee osteoarthritis, Symptomatic osteoarthritis, Radiographic osteoarthritis, Meta-analysis

## Abstract

**Objective:**

This systematic review and meta-analysis aimed to evaluate the risk of all-cause mortality in patients with knee osteoarthritis (OA).

**Design:**

Comprehensive searches were conducted in PubMed, Embase, and the Cochrane Library on September 01, 2024. The review included cohort studies reporting risk estimates of all-cause mortality in knee OA patients compared to those without knee OA. Using a random-effects model, the pooled hazard ratios (HRs) were calculated. Subgroup analyses were performed according to the classification of knee OA, including radiographic knee OA only, symptomatic knee OA only, and radiographic and symptomatic knee OA.

**Results:**

A total of 15 cohort studies involving 1,023,799 participants were included in the systematic review, with 14 studies remaining for the meta-analysis. The meta-analysis revealed that knee OA patients had an increased risk of all-cause mortality compared to those without knee OA (pooled HR: 1.21; 95% confidence interval [CI]: 1.02, 1.45). Subgroup analyses indicated the mixed results, including radiographic knee OA only (pooled HR: 1.11; 95% CI: 0.97, 1.26), symptomatic knee OA only (pooled HR: 1.07; 95% CI: 0.80, 1.43), and radiographic and symptomatic knee OA (pooled HR: 1.58; 95% CI: 1.20, 2.07).

**Conclusions:**

This meta-analysis supports an association between knee OA and an increased risk of all-cause mortality, with a particularly pronounced risk observed in radiographic and symptomatic knee OA patients. Further research is needed to determine if OA at other sites also correlates with a higher risk of all-cause mortality.

## 1 Introduction

Osteoarthritis (OA) can occur in any joint, such as the spine, hip, or hand, and it most commonly impacts the knee [1]. Current management strategies for knee OA primarily focus on symptomatic relief, which may involve weight loss intervention, exercise therapy, topical or oral non-steroidal anti-inflammatory drugs (NSAIDs), and intraarticular injection with glucocorticoid or hyaluronic acid [2,3]. For patients with advanced knee OA or uncontrolled knee pain, joint replacement surgery for knee is considered a cost-effective option to improve quality of life and alleviate pain [4]. However, individuals undergoing total knee replacement are susceptible to post-operation complications, such as pulmonary embolism, sepsis, pneumonia, and even death [5,6]. In addition, patients with OA often experience a high prevalence of comorbidities, such as dyslipidemia, hypertension, depression, diabetes, cardiovascular disease, and peptic ulcer disease [7]. This burden of comorbidities may contribute to an increased risk of premature death among knee OA patients. Also, a systematic review and meta-analysis has shown that individuals with disabilities face a risk of all-cause mortality that is 2.24 times higher compared to those without disabilities [8]. Given that knee OA is a major cause of disability [9], numerous studies have emerged to investigate the association between knee OA and all-cause mortality.

One systematic review and meta-analysis found no significant difference in the risk of all-cause mortality in either symptomatic OA or radiographic OA with high heterogeneity across the included prospective cohort studies [10]. Another meta-analysis of individual participant-level data revealed that patients with OA-related knee pain or those with both OA-related knee pain and radiographic knee OA had higher mortality risks compared to individuals with neither OA-related knee pain nor radiographic knee OA [11]. However, no increased risk was observed in patients with radiographic knee OA alone [11]. Recent observational studies have reported conflicting results regarding the association between all-cause mortality and knee OA [[12], [13], [14], [15], [16], [17]]. Thus, the relationship between knee OA and the risk of all-cause mortality remained largely unclear. This systematic review and meta-analysis aimed to clarify this association by analyzing the risk of all-cause mortality in knee OA patients.

## 2 Methods

A systematic review and meta-analysis was conducted following the Preferred Reporting Items for Systematic Reviews and Meta-analyses (PRISMA) guidelines [18]. The study protocol was registered with PROSPERO (registration number: CRD42024584087).

### 2.1 Search strategy

Two investigators (PEK and AK) performed searches on PubMed, Embase, and the Cochrane Library from their respective inception to September 01, 2024. The detailed search strategy was described in Supplementary file. No restriction was applied regarding study design, language, or time period. Additionally, a snowballing approach was utilized, which involved manually reviewing relevant reviews, bibliographic references of the included studies, and articles citing these studies to identify more related studies.

### 2.2 Study selection

Studies were included if they reported data on odds ratio (OR), relative risk, standardized mortality ratio, incidence rate ratio, or hazard ratio (HR) for all-cause mortality and met the following criteria: (A) Observational studies aimed to investigate the association of risk of all-cause mortality with knee OA. (B) The study subjects were people with knee OA, but studies exclusively involving patients who had undergone knee replacement surgery were excluded. (C) The control group consisted of individuals without OA or the general population. (D) Only cohort studies were included, as interventional studies could not be conducted in this clinical setting. Case-control studies, cross-sectional studies, conference abstracts, review articles, editorials, letter to the editors, and unavailable full-texts were excluded.

Two investigators (PEK and AK) independently assessed the eligibility of the articles and retrieved the included studies, based on the criteria. Disagreements were resolved by discussion.

### 2.3 Data extraction and quality assessment

Data were independently extracted by two investigators (PEK and AK) from the selected studies, including study design, the name of the first author, country, publication year, number of patients in the study group and control group, definition of knee OA, covariates used for adjustment, and risk estimates (OR, relative risk, standardized mortality ratio, incidence rate ratio or HR) with 95% confidence intervals (CIs). Two investigators (PEK and AK) used the Newcastle-Ottawa Scale (NOS) to evaluate the risk of bias in the included studies [19].

### 2.4 Statistical analysis and time-to-event data meta-analysis

Time-to-event data meta-analyses were conducted by using a logarithmic transformed inverse variance-weighted approach. Studies reporting HRs for all-cause mortality as an outcome was included in the meta-analyses. When multiple risk estimates were reported, we adopted the risk estimates that were adjusted for the most comprehensive set of confounders. Results were reported as pooled HRs with their corresponding 95% CIs for all-cause mortality among knee OA patients. Restricted maximum likelihood random-effects models were employed to account for expected clinical heterogeneity [20]. Subgroup analyses were performed according to the classification of knee OA, including radiographic knee OA only, symptomatic knee OA only, and radiographic and symptomatic knee OA. The radiographic knee OA only was defined as having Kellgren-Lawrence (KL) score of 2 or higher in one knee [21]. The radiographic and symptomatic knee OA was defined as having radiographic knee OA and knee pain in the same knee. The “symptomatic knee OA only” subgroup comprised studies that did not assess the KL score of knee OA. If the study provided graphical displays without numerical data, we used WebPlotDigitizer (version 5.2; https://automeris.io/) to extract the graphed data for risk estimates.

To detect publication bias, a funnel plot was created and visually inspected if more than 10 studies were included in the meta-analysis [22]. A one-study removed sensitivity analysis across the entire meta-analysis would be performed. This analysis would involve recalculating the results iteratively by removing one study at a time. Furthermore, sensitivity analyses were conducted exclusively on the studies with any adjustment of body mass index (BMI), the use of NSAIDs, physical activity, or comorbidities. Subgroup analyses were also performed based on gender (male/female) and age (≥65 years old vs ​< ​65 years old).

Review Manager (RevMan) 5.4 software (Cochrane Collaboration) was used to perform all statistical analyses. The degree of heterogeneity across the included studies was evaluated using the I-square statistic. I-square values below 40% indicated low heterogeneity between studies, values between 40% and 75% indicated moderate heterogeneity, and values exceeding 75% indicated considerable heterogeneity. Statistically significant difference was determined with a p-value of less than 0.05.

## 3 Results

### 3.1 Characteristics of included studies

**Fig. 1:**
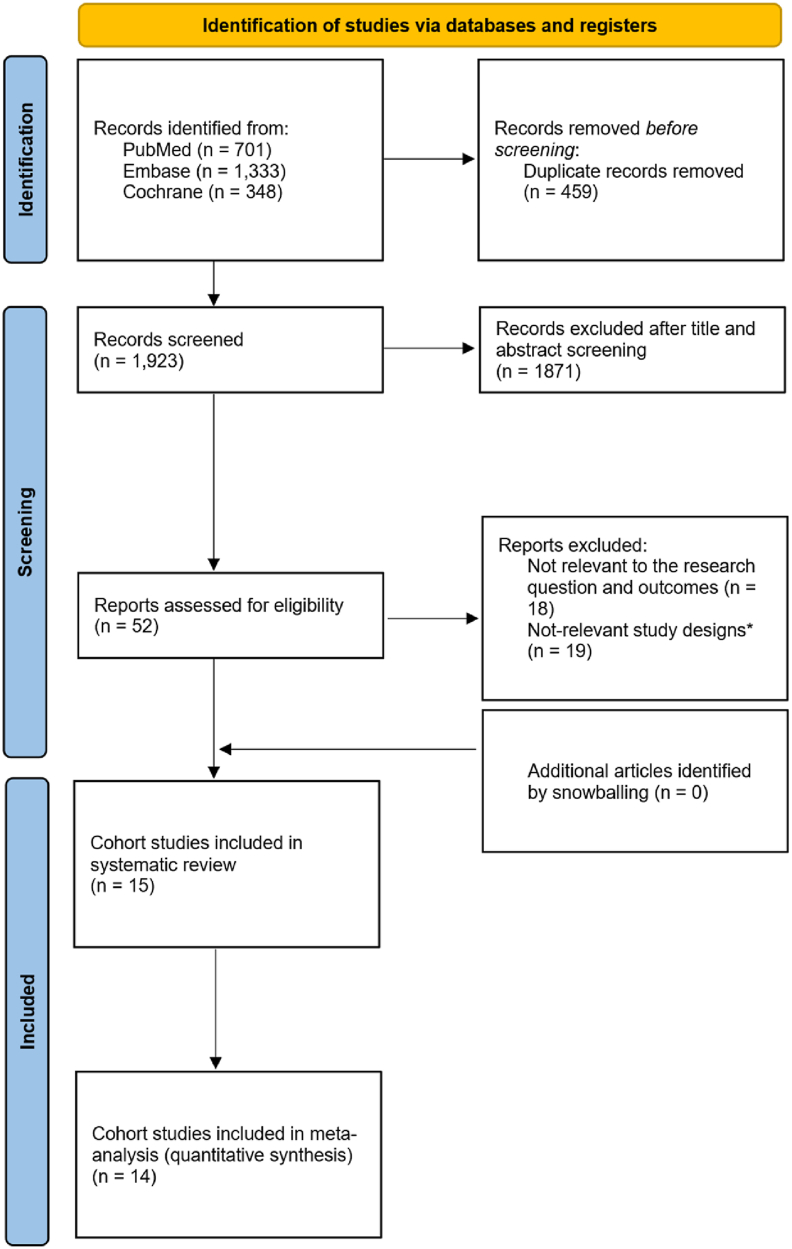
Preferred Reporting Items for Systematic Reviews and Meta-analyses (PRISMA) flowchart. ∗Not-relevant study designs included those that were not cohort studies, such as interventional studies, conference abstracts, case-control studies, cross-sectional studies, editorials, review articles, and letters to the editor.

A total of 2382 studies were initially retrieved from the three databases. After excluding duplicates and screening based on the title and abstract, 52 studies were selected for further assessment. Based on the inclusion and exclusion criteria, 15 cohort studies were included in the systematic review [[12], [13], [14], [15], [16], [17],[23], [24], [25], [26], [27], [28], [29], [30], [31]]. Of these, 14 studies were retained for the meta-analysis [[12], [13], [14], [15], [16], [17],[24], [25], [26], [27], [28], [29], [30], [31]]. The PRISMA 2020 flow diagram illustrating the study selection process is shown in Fig. 1, and a summary of the characteristics of the included studies is provided in Table 1 [[12], [13], [14], [15], [16], [17],[23], [24], [25], [26], [27], [28], [29], [30], [31]].

**Table 1:** Characteristics of the included studies.

Source	Country	Study design	Exposed group	Control group	Covariates adjusted for	Definition of knee OA
Tsuboi et al., 2011 [23]	Japan	Cohort study	244 knee OA cases	545 controls without knee OA	Age, sex, BMI, and lifestyle including habits of smoking, exercise, and drinking.	Knee OA was defined as having a KL grade of 2 or higher.
Liu et al., 2015a [24]	China	Cohort study	63 symptomatic knee OA patients and 181 radiographic knee OA patients.	962 controls without symptomatic knee OA and 844 pts controls without radiographic OA.	Age, sex, BMI, education, income level, comorbidities, and levels of occupational physical activity.	Tibiofemoral radiographic OA was defined as KL score of ≥2 in the tibiofemoral joint. Patellofemoral radiographic OA was identified by either an osteophyte of grade 2 or higher, or JSN of grade 2 or higher with a concurrent grade 1 osteophyte in the patellofemoral joint. Radiographic OA was defined as tibiofemoral or patellofemoral ROA. Symptomatic OA was defined as the coxistence of pain and radiographic OA in the same knee.
Liu et al., 2015b [25]	Netherlands	Cohort study	383 cases with symptomatic primary OA. The number of symptomatic primary knee OA was not reported.	Those without knee OA. The number of controls was not reported.	Age and sex.	Symptomatic primary knee OA was defined as the diagnosis by rheumatologists, general practitioners, and orthopaedic surgeons.
Kluzek et al., 2016 [26]	United Kingdom	Cohort study	57 patients with symptomatic knee OA and 64 patients with radiographic knee OA only.	416 controls with neither knee pain nor radiographic knee OA.	Age, total cholesterol, HDL, smoking, cholesterol, systolic blood pressure, antihypertension medication, occupation, BMI, past physical activity, HRT use, glucose levels, current/previous CVD disease, and the use of NSAIDs except from aspirin.	A KL score of ≥2 in at least one knee was classified as radiographic knee OA. The definition of knee pain was determined through a self-administered questionnaire, which asked respondents if they had experienced knee pain in the past month. Symptomatic knee OA was defined as the presence of both knee pain and radiographic knee OA.
Turkiewicz et al., 2016 [27]	Sweden	Cohort study	51,939 knee OA patients.	524,136 controls who were general population individuals seeking healthcare.	Sex, income, highest level of education achieved, residential area, marital status, comorbidities, and year of first healthcare visit.	Knee OA was based on ICD-10 diagnostic codes.
Veronese et al., 2016 [28]	Italy	Cohort study and systematic review and meta-analysis	140 knee OA patients.	241 controls without OA.	Age, sex, BMI, educational level, income, alcohol drinking, comorbidity, physical activity level, medication use, smoking status, and activities of daily living, geriatric depression scale, mini-mental state, and geriatric nutrition risk index scores.	The presence of knee OA was confirmed through a review of medical records, clinical evaluation, previous X-ray reports, and final confirmation by a rheumatologist.
Liu et al., 2017 [30]	China	Cohort study	63 symptomatic knee OA cases.	962 controls without symptomatic knee OA.	Age, sex, education, BMI, level of income, comorbidities, and level of daily physical activity.	Tibiofemoral radiographic OA was determined through a KL score of 2 or higher in the tibiofemoral joint. Patellofemoral radiographic OA was defined as the presence of an osteophyte of grade 2 or above, or JSN of grade 2 or above along with a grade 1 osteophyte in the patellofemoral joint. Symptomatic knee OA was determined when both pain and either tibiofemoral or patellofemoral radiographic OA were observed in the same knee.
Kasai et al., 2017 [29]	Japan	Cohort study	154 knee OA cases	447 controls without knee OA	Age, sex, and BMI.	Knee OA was defined as having a KL grade of 2 or higher. The presence of knee pain was identified through a questionnaire.
Mendy et al., 2018 [12]	United States	Cohort study	3428 self-reported OA cases and 2589 radiographic OA cases.	39,473 controls who denied having arthritis and 874 controls without arthritis.	Age, sex, race, smoking, BMI, poverty income ratio, physical activity, hypertension, diabetes, stroke, and myocardial infarction.	Self-reported OA was identified by asking few questions to the participants. Radiographic OA was defined as KL score was ≥2 in the knee.
Cleveland et al., 2019 [13]	United States	Cohort study	1874 radiographic knee OA cases and 1177 symptomatic knee OA cases.	2308 controls without radiographic knee OA and 3005 controls without symptomatic knee OA.	Age, sex, race, knee injury, education, non-steroidal anti-inflammatory drugs, smoking, alcohol use, cancer, obesity, diabetes, hypertension, cardiovascular disease, liver disease, physical activity, and depression.	KL score not less than 2 in the knee was classified as having radiographic knee OA. Severe radiographic knee OA was defined as those with KL score ≥3. Cases of radiographic knee OA that self-reported pain in the same knee as the radiographic knee OA were classified as symptomatic knee OA.
Yang et al., 2019 [14]	Including 12 countries (Austria, Belgium, Denmark, France, Greece, Germany, Sweden, Spain, Switzerland, Italy, Israel, and Netherlands).	Cohort study	The number of symptomatic knee OA cases was 1529 cases in the ELSA, 1710 cases in the SHARE, 1166 cases in the KLoSA, and 418 cases in the IFLS.	The number of those without symptomatic knee OA was 9322 cases in the ELSA, 26,823 cases in the SHARE, 8754 cases in the KLoSA, and 9800 cases in the IFLS.	Age, sex, BMI, smoking status, education level, alcohol use, diabetes, hypertension, cancer, stroke, chronic lung diseases, and heart diseases.	The participants were asked whether they had been troubled by joint pain frequently lately and had a diagnosis of arthritis.
Wang et al., 2021 [31]	United States	Cohort study	1117 radiographic knee OA cases and 1387 symptomatic knee OA cases.	1139 controls with neither knee pain nor radiographic knee OA.	Age, sex, BMI, race, weekly alcohol consumption, education level, history of knee injury, smoking habits, depressive symptoms, widespread pain, and comorbidities.	A knee with a KL grade of ≥2 was classified as radiographic knee OA. Knee pain was determined based on self-reported symptoms, specifically if the participant experienced pain, aching, or stiffness in the knee for most of the time over at least one month in the past year. The knee pain combined with radiographic knee OA was used to define symptomatic knee OA.
Oh et al., 2023 [15]	Korea	Cohort study	2340 radiographic knee OA cases without knee pain and 1346 symptomatic knee OA cases.	5580 controls without both radiographic knee OA and knee pain.	Age, sex, hypertension, obesity, diabetes, household income, total cholesterol, stroke, and myocardial infarction.	Radiographic knee OA was defined as a knee joint KL grade 2 or higher. Knee pain was defined as experiencing knee pain for more than 30 days in the past three months. Symptomatic knee OA was defined as having both radiographic knee OA and knee pain simultaneously.
Swain et al., 2023 [17]	United Kingdom	Cohort study	53,982 knee OA cases.	59,351 controls without knee OA.	Age, sex, BMI, alcohol, smoking, Elixhauser comorbidity index, and comorbidities at baseline.	Knee OA was determined using a standard clinical coding system commonly employed in United Kingdom general practice.
Park et al., 2023 [16]	Korea	Cohort study	7572 knee OA cases.	193,894 controls without knee OA.	Age, sex, BMI, income, dyslipidemia, hypertension, smoking, alcohol consumption, glucose levels, physical activity, and glomerular filtration rate.	Knee OA was identified using the ICD-10 code for knee OA (M17) or any code for OA at any site when accompanied by a knee X-ray procedure on the same claim

BMI: body mass index; CVD: cardiovascular disease; ELSA: English Longitudinal Study of Aging; HDL: high-density lipoprotein cholesterol; HRT: hormone replacement therapy; ICD-10: International Classification of Diseases 10th Revision; IFLS: Indonesian Family Life Survey; JSN: joint space narrowing; KL grade; Kellgren-Lawrence grade; KLoSA: the Korean Longitudinal Study of Aging; NSAIDs: non-steroidal anti-inflammatory drugs; OA: osteoarthritis; SHARE: Survey of Health, Aging and Retirement in Europe.

### 3.2 Risk of all-cause mortality among knee OA patients

**Fig. 2:**
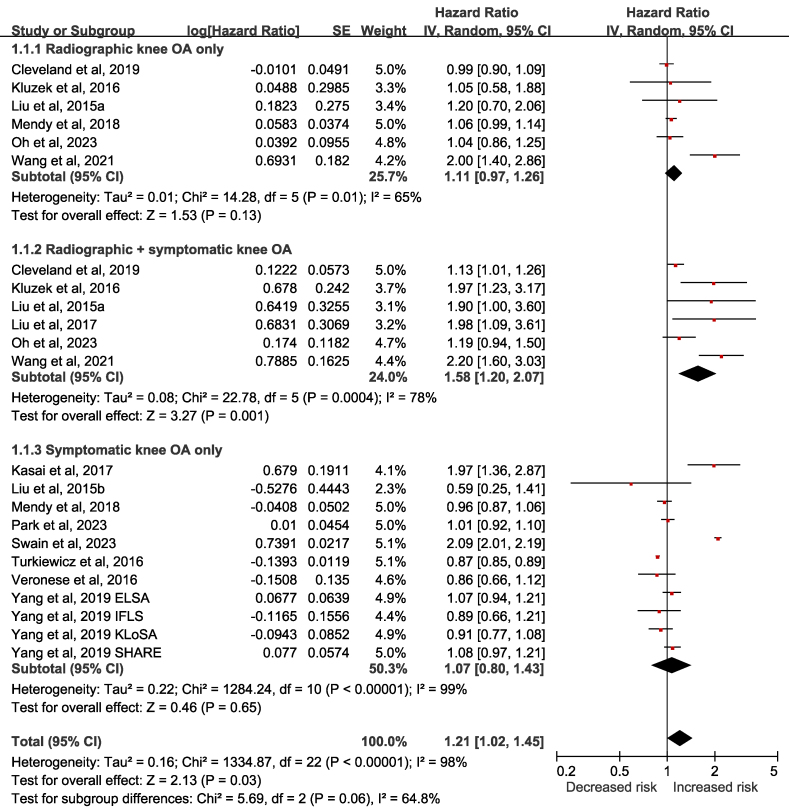
Forest plot on the association between risk of all-cause mortality and knee osteoarthritis (OA) in cohort studies. Subgroup analyses based on the classification of knee OA were performed. CI: confidence interval; ELSA: English Longitudinal Study of Aging; IFLS: Indonesian Family Life Survey; IV: inverse variance; KLoSA: the Korean Longitudinal Study of Aging; OA: osteoarthritis; SE: standard error; SHARE: Survey of Health, Aging and Retirement in Europe.

15 cohort studies, involving a total of 1,023,799 participants, were conducted to investigate the risk for all-cause mortality in knee OA patients compared to patients without knee OA or general population [[12], [13], [14], [15], [16], [17],[23], [24], [25], [26], [27], [28], [29], [30], [31]]. One study reported an increased risk of death after 10 years in the patients with knee OA (OR: 2.316; 95% CI: 1.412, 3.801), compared to those without [23]. Because the HR for all-cause mortality was not provided, this study was not included in the meta-analysis. The other 14 cohort studies had reported the HRs for all-cause mortality in the knee OA patients [[12], [13], [14], [15], [16], [17],[24], [25], [26], [27], [28], [29], [30], [31]]. In Fig. 2, the meta-analysis showed an increased risk for all-cause mortality in knee OA patients (pooled HR: 1.21; 95% CI: 1.02, 1.45; p value ​= ​0.03) with considerable heterogeneity (I^2^ ​= ​98%) across these 14 cohort studies. The subgroup analyses according to the classification of knee OA revealed mixed results. An increased risk for all-cause mortality was detected in patients with radiographic and symptomatic knee OA (pooled HR: 1.58; 95% CI: 1.20, 2.07; p value ​= ​0.001; I^2^ ​= ​78%), but not in the patients with radiographic knee OA only (pooled HR: 1.11; 95% CI: 0.97, 1.26; p value ​= ​0.13; I^2^ ​= ​65%) and symptomatic knee OA only (pooled HR: 1.07; 95% CI: 0.80, 1.43; p value ​= ​0.65; I^2^ ​= ​99 ​%).

### 3.3 Risk of bias and publication bias of included studies

**Fig. 3:**
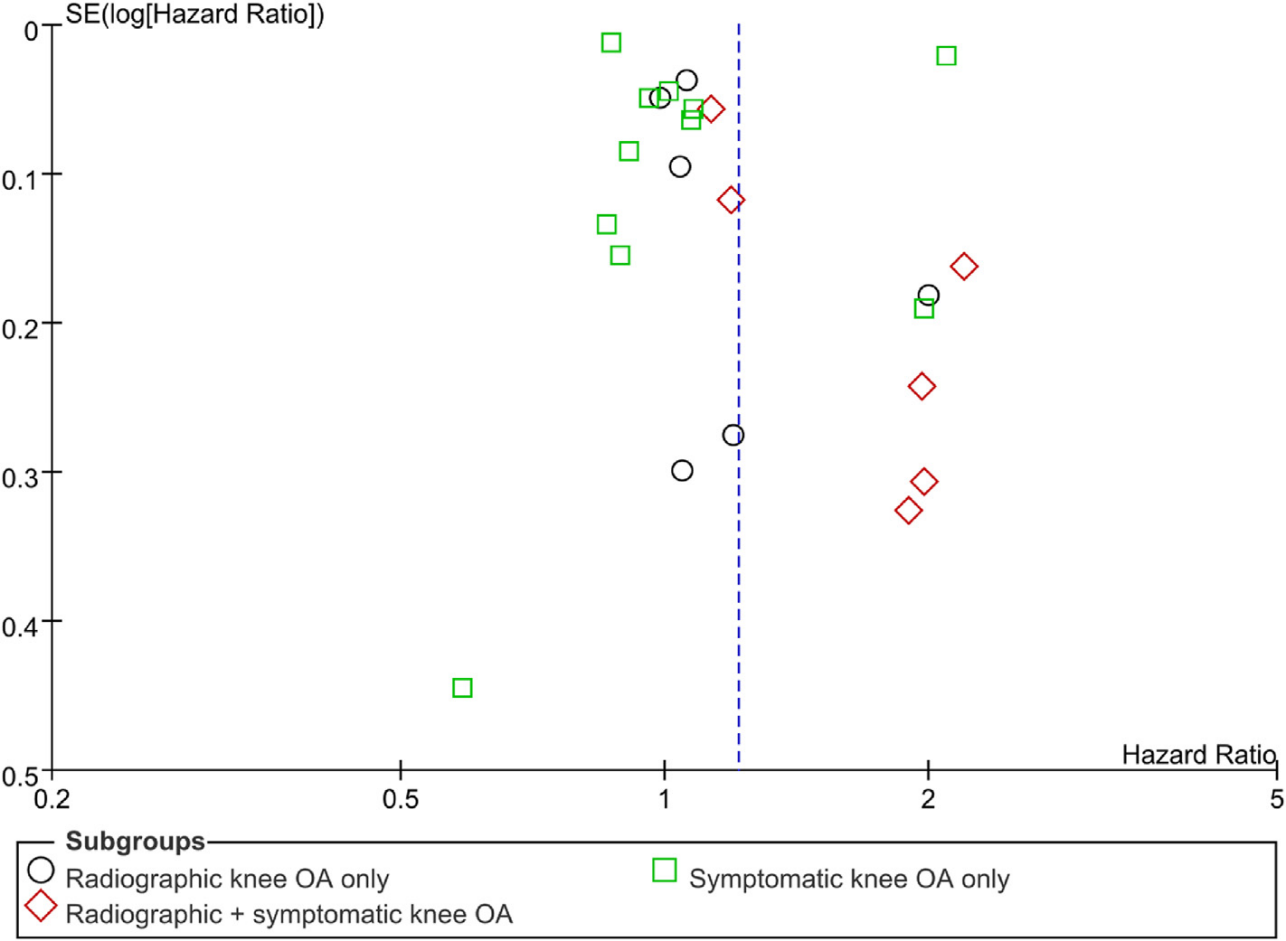
Funnel plot of effect sizes for the included cohort studies in the meta-analysis. This funnel plot illustrates the hazard ratios of cohort studies in relation to their standard errors. Published cohort studies are represented by different markers: dark circles for studies reporting radiographic knee osteoarthritis (OA) only, green squares for studies reporting symptomatic knee OA only, and red diamonds for studies reporting radiographic and symptomatic knee OA. The dotted line represents the expected results of cohort studies based on the estimated underlying effect size. SE: standard error; OA: osteoarthritis.

Risk of bias of the included cohort studies was shown in Table 2. All studies indicated a low risk of bias with NOS scores ranging from seven to nine [[12], [13], [14], [15], [16], [17],[23], [24], [25], [26], [27], [28], [29], [30], [31]]. The funnel plot was presented in Fig. 3. There was no obvious asymmetry of the funnel plot, which indicated a low risk of publication bias.

**Table 2:** Quality assessment of the included cohort studies.

Source	Quality of selection	Comparability	Outcomes of study participants
Tsuboi et al., 2011 [23]	★★★★	–	★★★
Liu et al., 2015a [24]	★★★★	★★	★★★
Liu et al., 2015b [25]	★★★★	–	★★★
Kluzek et al., 2016 [26]	★★★	★★	★★★
Turkiewicz et al., 2016 [27]	★★★★	–	★★★
Veronese et al., 2016 [28]	★★★★	★★	★★★
Liu et al., 2017 [30]	★★★★	★★	★★★
Kasai et al., 2017 [29]	★★★★	–	★★★
Mendy et al., 2018 [12]	★★★	★★	★★★
Cleveland et al., 2019 [13]	★★★★	★★	★★★
Yang et al., 2019 [14]	★★★	★★	★★★
Wang et al., 2021 [31]	★★★★	★★	★★★
Oh et al., 2023 [15]	★★★★	★★	★★★
Swain et al., 2023 [17]	★★★★	★★	★★★
Park et al., 2023 [16]	★★★★	★★	★★★

Stars (★) were awarded based on Newcastle-Ottawa Quality Assessment Form for Cohort Studies.

### 3.4 Sensitivity analysis

The one-study removed sensitivity analysis was performed as illustrated in Table 3. In the sensitivity analysis for knee OA, the pooled HRs ranged from 1.15 (95% CI: 0.96, 1.38; p value ​= ​0.13) to 1.27 (95% CI: 1.03, 1.57; p value ​= ​0.02). Some of them showed no significant difference in risk for all-cause mortality between the knee OA patients and the controls. In Table 4, the sensitivity analyses, which were limited to studies that adjusted for BMI or comorbidities, found a significant association between all-cause mortality and knee OA. However, this association was not significant when restricted to studies that adjusted for the use of NSAIDs or physical activity. In the subgroup analyses based on gender and age, most results did not show a significant increase in the risk of all-cause mortality among patients with knee OA. When limited to studies involving participants not younger than 65 years, a negative association between all-cause mortality and knee OA was observed (pooled HR: 0.89; 95% CI: 0.83, 0.95; p ​= ​0.001).

**Table 3:** The one-study removed sensitivity analysis for knee osteoarthritis (OA).

The omitted study	Pooled HR (95% CI)	p-value	I^2^
Cleveland et al., 2019 [13]	1.23 [1.01, 1.50]	0.04^a^	98
Kasai et al., 2017 [29]	1.19 [0.99, 1.42]	0.06	98
Kluzek et al., 2016 [26]	1.19 [0.99, 1.43]	0.06	98
Liu et al., 2015a [24]	1.19 [0.99, 1.43]	0.06	98
Liu et al., 2015b [25]	1.23 [1.03, 1.47]	0.02^a^	98
Liu et al., 2017 [30]	1.19 [1.00, 1.43]	0.05	98
Mendy et al., 2018 [12]	1.24 [1.01, 1.52]	0.04^a^	98
Oh et al., 2023 [15]	1.22 [1.01, 1.48]	0.04^a^	99
Park et al., 2023 [16]	1.22 [1.01, 1.48]	0.03^a^	98
Swain et al., 2023 [17]	1.12 [1.03, 1.22]	0.01^a^	87
Turkiewicz et al., 2016 [27]	1.23 [1.03, 1.47]	0.02^a^	97
Veronese et al., 2016 [28]	1.23 [1.03, 1.48]	0.02^a^	98
Wang et al., 2021 [31]	1.15 [0.96, 1.38]	0.13	98
Yang et al., 2019 b [14]	1.27 [1.03, 1.57]	0.02^a^	99

CI: confidence interval; HR: hazard ratio; OA: osteoarthritis.

a Significant overall effect at p-value <0.05.

b All four cohorts in the Yang et al., 2019 were excluded.

**Table 4:** Summary of risk estimates for the association between all-cause mortality and knee osteoarthritis (OA).

	The number of studies	Pooled HR (95% CI)	p-value	I^2^
Overall	14 [[12], [13], [14], [15], [16], [17], [24], [25], [26], [27], [28], [29], [30], [31]]	1.21 [1.02, 1.45]	0.03^a^	98
Studies with any adjustment of				
BMI	10 [[12], [14], [16], [17], [24], [26], [28], [29], [30], [31]]	1.31 [1.06, 1.62]	0.01^a^	98
The use of NSAIDs	2 [[13], [26]]	1.13 [0.95, 1.35]	0.18	69
Physical activity	7 [[12], [13], [16], [24], [26], [28], [30]]	1.05 [0.98, 1.14]	0.18	57
Comorbidities	12 [[12], [13], [14], [15], [16], [17], [24], [26], [27], [28], [30], [31]]	1.21 [1.01, 1.45]	0.04^a^	98
Gender				
Male	3 [[13], [16], [27]]	0.95 [0.81, 1.12]	0.56	85
Female	4 [[13], [16], [26], [27]]	1.06 [0.87, 1.30]	0.55	89
Age ≥65 years old	3 [[13], [16], [27]]	0.89 [0.83, 0.95]	0.001^a^	79
Age <65 years old	3 [[13], [16], [27]]	0.97 [0.80, 1.17]	0.73	91

BMI: body mass index; CI: confidence interval; HR: hazard ratio; NSAIDs: non-steroidal anti-inflammatory drugs; OA: osteoarthritis.

a Significant overall effect at p-value <0.05.

## 4 Discussion

The current systematic review and meta-analysis found that knee OA was associated with an increased risk of all-cause mortality. This association was particularly evident in patients with radiographic and symptomatic knee OA, but not in those with radiographic knee OA only or symptomatic knee OA only. Although the sensitivity analyses showed inconsistent results, a trend of increased risk of all-cause mortality in knee OA patients was observed.

Previous meta-analyses have investigated the association between OA and all-cause mortality [10,11,28,32]. One meta-analysis involving nine prospective cohort studies found no significant association between all-cause mortality and either radiographic or symptomatic OA [10]. However, the anatomic site of OA was not specified in this study [10]. Given that OA is a heterogeneous joint disease with varying management approaches depending on the affected site, subgroup analyses based on different OA locations are necessary [33]. For instance, walking disability and the use of NSAIDs have been proposed as mechanisms linking knee OA to mortality [30,31], highlighting the need for such subgroup analyses. A prospective cohort study with meta-analysis revealed no association between knee OA and all-cause mortality [28]. Another meta-analysis has found no association between knee OA and all-cause mortality after the integration of six cohort studies [32]. Conversely, an international meta-analysis of individual participant-level data including six community-based cohorts showed that all-cause mortality was associated with the presence of both knee pain and radiographic knee OA, but not associated with radiographic knee OA without knee pain, compared to those without radiographic knee OA and knee pain [11]. The risk estimate was attenuated after the adjustment of covariables, including age, sex, BMI, and comorbidities. Similarly, the present study demonstrated a higher risk of all-cause mortality in the patients with knee OA, particularly those with radiographic and symptomatic knee OA, but not in those with radiographic knee OA only or symptomatic knee OA only. The present study has several strengths compared to the previous meta-analyses. First, we included a total of 15 cohort studies to provide up-to-date evidence. Second, the use of NSAIDs and physical activity are potential factors linking knee OA to mortality. The present study found that when limited to studies adjusting for the use of NSAIDs or physical activity, a neutral effect of knee OA on all-cause mortality was observed. Furthermore, in the population aged 65 years and older, a protective effect of knee OA was noted. Future research is warranted.

Seven cohort studies reported on the risk of all-cause mortality in the patients with radiographic knee OA only [12,13,15,23,24,29,31]. The Osteoarthritis initiative (OAI) cohort study conducted in the United States reported a significant two-fold increase in the risk of all-cause mortality in the radiographic knee OA patients, but this study also indicated a lower mortality rate of 14.1 deaths per 1000 person-years compared to other studies [31]. Differences in findings may be attributed to varying comorbidity burdens. For example, two other cohort studies also conducted in the United States reported higher mortality rates [12,13]. In the National Health and Nutrition Examination Surveys (NHANES) cohort study, the radiographic knee OA patients had a mortality rate of 67 deaths per 1000 person-years [12]. Similarly, in the cohort study using data from the Johnston County Osteoarthritis Project, 845 out of 1874 radiographic knee OA patients had died at the study endpoint with a median follow-up time of 14.6 years [13]. By using modified Charlson comorbidity index, the mean comorbidity score at baseline was similar between the patients with radiographic knee OA and the controls in the OAI cohort study [31]. In the NHANES cohort study, the radiographic knee OA patients had a higher prevalence of diabetes, hypertension, and history of myocardial infarction or stroke, compared to the controls [12]. The comorbidities such as diabetes and cardiovascular diseases are the leading cause of death worldwide [34]. Future research is warranted to explore the effect of radiographic knee OA on the risk of all-cause mortality among patients with fewer comorbidities.

In terms of radiographic and symptomatic knee OA, five cohort studies reported the risk of all-cause mortality in knee OA, specifying either radiographic only or symptomatic knee OA [13,15,24,26,31]. All of these studies defined symptomatic knee OA as the presence of both radiographic knee OA and pain in the same knee. They generally indicated a trend towards a higher risk of all-cause mortality in patients with symptomatic knee OA compared to those with radiographic knee OA. The Genetic Arthrosis and Progression (GARP) cohort study did not find an association between all-cause mortality and symptomatic knee OA [25]. This study had a small sample size and only adjusted for age and sex. Additionally, one study using data from four international surveys of aging found no association between symptomatic knee OA and all cause-mortality [14]. The ascertainment of symptomatic knee OA relied on self-report data, which could lead to misclassification.

As for symptomatic knee OA only, the study found no association between knee OA and all-cause mortality [16]. On the contrary, a cohort study from Clinical Practice Research Datalink GOLD database found the positive association [17], while another cohort study found the negative association [27]. As these three cohort studies applied diagnostic code to identify the case of knee OA, misclassification might exist.

The mechanisms linking knee OA to all-cause mortality remain poorly understood. First, the Wuchuan Osteoarthritis study suggested that the effect of symptomatic knee OA was mainly mediated by walking disability and the use of NSAIDs, though these findings were not statistically significant due to a small sample size [30]. Disabilities or physical inactivity are known risk factors for all-cause mortality and cardiovascular disease [8,35,36]. Furthermore, long-term use of NSAIDs may lead to serious gastrointestinal, cardiovascular, or renal adverse events [37,38]. Several studies have proposed that knee pain, rather than radiographic knee OA, is a mortality risk factor. The Chingford cohort study demonstrated that knee pain without radiographic knee OA was associated with an increased risk of cardiovascular disease mortality, compared to those without both knee pain and radiographic knee OA [26]. A trend towards increased risk of all-cause mortality was also observed in this study albeit statical significance was not reached [26]. Another cohort study found no association between knee pain alone and either all-cause mortality or cardiovascular disease mortality, possibly due to the limited sample size with the only 71 knee pain patients included [15]. The meta-analysis of individual participant-level data revealed that knee OA-related pain was positively associated with all-cause mortality [11]. Therefore, radiographic and symptomatic knee OA may lead to increased NSAIDs use and associated adverse effects, partially explaining the link between radiographic and symptomatic knee OA and all-cause mortality. Supporting this concept, the cohort study that defined severe radiographic knee OA as having KL grade not less than 3 revealed that severe radiographic knee OA had a neutral effect on all-cause mortality (HR: 0.96; 95% CI: 0.86, 1.08) [13]. This implies that it is not the structural changes associated with knee OA, but rather the symptoms of knee OA, that are more closely linked to all-cause mortality.

There were several strengths in the present study. First, the present study includes diverse patient populations from various regions, including China, Italy, Japan, Korea, the United States, the United Kingdom, the Netherlands, and Sweden, enhancing external validity. Second, we have performed the subgroup analyses for the classification of knee OA, and found that all-cause mortality was associated with radiographic and symptomatic knee OA but not with radiographic knee OA only or symptomatic knee OA only. That said, there are some limitations. First, considerable heterogeneity was present in the association between knee OA and all-cause mortality. Hence, we applied a random-effects model, and sensitivity and subgroup analyses were performed, albeit the considerable heterogeneity still existed. Therefore, the findings of the present study should be interpreted cautiously. Second, the included studies were all cohort studies and residual confounders were inevitable. However, interventional study designs are not feasible in this clinical setting. Third, the present study focuses on the risk of all-cause mortality in knee OA, and future research is warranted to explore the relationship between cause-specific mortality and OA of knee or other sites.

In conclusion, an increased risk of all-cause mortality is observed in the knee OA patients, especially those with radiographic and symptomatic knee OA. Despite the considerable heterogeneity in the current literature, clinicians need to consider the radiographic and symptomatic knee OA to be a potential risk factor for premature death.

## Author contributions

**Pei-En Kao**: Conception and design of the study, acquisition of data, analysis and interpretation of the data, drafting the article. **Amy Ker**: Acquisition of data, analysis and interpretation of the data, drafting the article. All authors have agreed to the final version of the manuscript to be published.

## Role of the funding source

No funding was provided for this study.

## Declaration of competing interest

All authors declare no conflict of interest.
