# Supplementary material for: Risk of all-cause mortality in patients with knee osteoarthritis: A systematic review and meta-analysis of cohort studies
Source: Osteoarthr Cartil Open. 2024 Nov 8;7(1):100541. doi: 10.1016/j.ocarto.2024.100541 (PMC11616512; doi:10.1016/j.ocarto.2024.100541)
Supplement: Multimedia component 1 [file mmc1.docx]

**Supplementary file**

**eTable 1.** Detailed search strategy retrieved on 2024.09.01

| PubMed search strategy | |
| --- | --- |
| 1 | "osteoarthritis, knee"[MeSH Terms] OR "knee oa"[Text Word] OR "knee osteoarthri*"[Text Word] OR "osteoarthritis of the knee"[Text Word] OR "osteoarthritis of knee"[Text Word] |
| 2 | "mortality"[MeSH Terms] OR "mortal*"[Text Word] OR "all cause mortality"[Text Word] OR "death*"[Text Word] OR "dead"[Text Word] |
| 3 | #1 AND #2 |
| Embase search strategy | |
| 1 | ('knee osteoarthritis'/exp OR 'knee oa' OR 'knee osteoarthri*' OR 'osteoarthritis of the knee' OR 'osteoarthritis of knee') AND ('mortality'/exp OR mortal* OR 'all cause mortality' OR death* OR dead) |
| Cochrane Library search strategy | |
| 1 | MeSH descriptor: [Osteoarthritis, Knee] explode all trees |
| 2 | (knee OA) |
| 3 | knee osteoarthri* |
| 4 | Osteoarthritis of the Knee |
| 5 | Osteoarthritis of Knee |
| 6 | #1 OR #2 OR #3 OR #4 OR #5 |
| 7 | MeSH descriptor: [Mortality] explode all trees |
| 8 | mortal* |
| 9 | all-cause mortality |
| 10 | death* |
| 11 | dead |
| 12 | #7 OR #8 OR #9 OR #10 OR #11 |
| 13 | #6 AND #12 |
